# Untargeted GC-IMS Metabolomics of Wound Headspace for Bacterial Infection Biomarker Discovery

**DOI:** 10.3390/metabo16040272

**Published:** 2026-04-17

**Authors:** Yanyi Lu, Bowen Yan, Lin Zeng, Bangfu Zhou, Ruoyu Wu, Xiaozheng Zhong, Qinghua He

**Affiliations:** 1 Department of Field Medical Equipment, Daping Hospital, Army Medical University, Chongqing 400042, China; luyanyi@tmmu.edu.cn (Y.L.); yanbowen@tmmu.edu.cn (B.Y.); lzeng118@tmmu.edu.cn (L.Z.); zhoubangfu@tmmu.edu.cn (B.Z.); wuruoyu@tmmu.edu.cn (R.W.); 2 War Trauma Medical Center, Daping Hospital, Army Medical University, Chongqing 400042, China

**Keywords:** untargeted metabolomics, volatile metabolite, metabolic biomarkers, wound bacterial infection, gas chromatography–ion mobility spectrometry, rapid identification

## Abstract

**Highlights:**

**What are the main findings?**

- The combination of GC-IMS and machine learning can successfully identify the presence of infection and *Escherichia coli* infection.
- Eight characteristic features that can be used to identify bacterial infection and six characteristic features that can be used to identify *Escherichia coli* infection were selected. Volatile metabolites such as 3-methyl-1-butanol, 2-methyl-1-butanol, and ethyl hexanoate were successfully annotated as potential biomarkers for bacterial infection.

**What are the implications of the main findings?**

- The findings provide a promising new strategy for the rapid diagnosis of wound infections at the bedside, potentially reducing antimicrobial overuse and improving patient prognosis through more timely and targeted antibiotic therapy.
- The potential volatile metabolic biomarkers of wound infection discovered in this study can provide new insights into the study of metabolic volatiles in wounds.

**Abstract:**

**Background/Objectives:** Wound infections cause significant morbidity, yet current diagnostics rely on time-consuming microbial culture. Volatile organic compounds (VOCs) from bacterial metabolism offer potential for early diagnosis. This study aimed to validate the volatile metabolites profiled by gas chromatography–ion mobility spectrometry (GC-IMS) combined with machine learning for rapid identification of wound infections and certain bacterial infections. **Methods:** Headspace of clinical wound samples were analyzed using GC-IMS. Volatile metabolite profiles were compared between infected and non-infected groups and between *Escherichia coli* (*E. coli*)-positive and negative samples. Partial least squares discriminant analysis (PLS-DA) and Mann–Whitney U test were used for preliminary screening with variable importance in projection (VIP) > 1 and *p*-value < 0.05. Three machine learning algorithms, namely support vector machine (SVM), logistic regression (LR), and random forest (RF), were trained on the selected features for classification, using 5-fold cross-validation with 10 repeated runs. Model performance was assessed using key evaluation metrics, including accuracy, sensitivity, specificity, the area under the curve (AUC) and feature importance ranking to identify the most relevant biomarkers. **Results:** A total of 19 volatile metabolites associated with clinical wound samples were identified. The RF model achieved 90.15% sensitivity and 0.91 AUC for bacterial infection detection. For *E. coli* identification, LR reached 85.35% sensitivity and 0.89 AUC. Potential volatile metabolic biomarkers including elevated 3-methyl-1-butanol, 2-methyl-1-butanol, and ethyl hexanoate for identifying bacterial infection were selected through the cross-validation results of the three algorithms. **Conclusions:** Untargeted metabolomics by GC-IMS effectively captures infection-specific volatile metabolic signatures in complex wound samples. Integration with machine learning enables rapid, high-accuracy diagnosis of bacterial infections and *E. coli* identification at point of care. This approach addresses clinical metabolomics translational challenges by providing a portable and cost-effective method, potentially reducing antibiotic misuse through more timely and targeted therapy.

## 1. Introduction

A wound impairs the skin barrier, allowing microorganisms to invade and colonize, and systemic infection may develop if the infection is not properly controlled [1]. The increasing prevalence of multidrug resistance has increased the incidence and mortality of wound infections. Early identification of pathogenic bacteria, followed by timely targeted antibiotic therapy, has great significance for patient prognosis [2,3]. Clinically available microbial identification methods, either phenotypic (chromogenic tests, biochemical tests, and staining) or genotypic (polymerase chain reaction, DNA microarray, genome sequencing, and matrix-assisted laser desorption/ionization time-of-flight mass spectrometry), typically require hours to days, which is incompatible with the urgent clinical needs of critically ill patients [4,5]. Currently, patients with wound infections are often given empirical treatment with broad-spectrum antibiotics before targeted anti-infection therapy. This may lead to increased antibiotic resistance, adverse reactions, and prolonged hospital stay, which translate to higher costs and mortality [6,7]. Therefore, it is crucial to quickly identify the microorganisms infecting a wound early, so that appropriate targeted treatment can be selected [8].

Metabolomics is the systematic analysis of the complete set of small-molecule metabolites (the metabolome) within a biological system to investigate dynamic biochemical processes and their associations with physiological or pathological states and has been widely applied in the field of disease biomarker discovery. It has been demonstrated that volatile metabolites metabolized by bacteria can be used for rapid identification of pathogens, yielding results within minutes to tens of minutes [9]. Bacterial volatile metabolites may provide a new feasible solution for early diagnosis and monitoring of wound infections. Volatile metabolites can be detected by chromatographic methods and volatile organic compounds (VOCs) sensor-based methods [10,11,12]. VOCs sensors detect certain compounds and produce quantifiable signals, offering a practical and portable means for VOCs analysis; however, the signal can hardly translate back to specific compounds. Among chromatographic methods, gas chromatography–mass spectrometry (GC-MS) is widely recognized as the gold standard for the analytical detection of volatile organic compounds. This technique separates compounds with a chromatographic column, ionizes them, and identifies the compounds based on their distinct mass-to-charge ratios.

GC-MS has been used to analyze the headspace of bacterial biofilm in human ex vivo cutaneous wound models and showed significant correlation between the VOC profiles and phases of biofilm formation [13]. Analysis of volatile metabolites in human wounds by GC-MS revealed unique VOC “fingerprints” of different pathogens, providing important experimental evidence and a biomarker library for the development of non-invasive, rapid bedside diagnostic technologies for infections [14,15]. Since GC-MS is expensive, large in size and requires highly specialized skills to operate, this prevents it from being portable.

For bedside care applications, ion mobility spectrometry (IMS) emerges as the most promising candidate because it offers a combination of affordability, a compact footprint, and reliability [6,16]. Moreover, gas chromatography–ion mobility spectrometry (GC-IMS) combines the pre-separation function of gas chromatography (GC) with the high sensitivity of ion mobility spectrometry (IMS) and achieves a detection limit to parts-per-billion level. At present, GC-IMS has been widely used to detect trace-amount analytes in food, environment, traditional Chinese medicine and bacterial cultures [17,18,19,20,21]. Using GC-IMS to detect bacterial volatile metabolites and thus rapidly identify wound-infecting bacteria could be promising. Volatile metabolite profile of various bacterial cultures and animal infection model by GC-IMS combined with machine learning demonstrated its rapid identification ability [22,23,24,25]. Our previous research showed that GC-IMS analysis of the headspace of bacterial cultures can identify bacteria in single and mixed bacterial cultures of three common wound infection bacteria [26]. However, the volatile metabolites used to identify bacteria depend on the culture medium and environment, and the real wound infection is much more complex. Direct detection of VOCs from wound swabs using GC-IMS, as opposed to bacterial-culture-derived VOCs, enabled the discrimination of infected versus uninfected clinical samples with high accuracy (sensitivity 100%, specificity 88%) through pattern recognition algorithms [27].

Further elucidation of volatile metabolic biomarkers specific to distinct infections detected by GC-IMS is warranted. This study employed GC-IMS to analyze the headspace VOCs directly from clinical wound samples to discover volatile metabolic biomarkers by GC-IMS combined with machine learning for identifying infection or even the presence of a specific bacterial infection. This study shall provide new insights for the volatile metabolites detected by GC-IMS in rapid identification of bacterial infections in clinical wounds.

## 2. Materials and Methods

### 2.1. Sample Collection and Study Design

**Figure 1:**
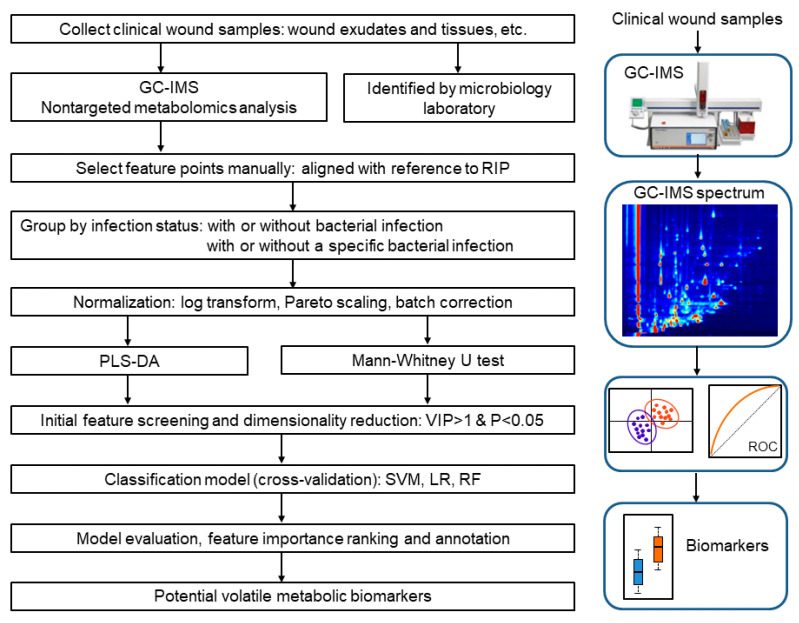
Research design and data analysis workflow. GC-IMS, gas chromatography–ion mobility spectrometry; PLS-DA, partial least squares discriminant analysis; VIP, variable importance in projection; SVM, support vector machine; LR, logistic regression; RF, random forest; ROC, receiver operating characteristic.

All wound samples were collected at War Trauma Medical Center, Daping Hospital, Army Medical University, Chongqing, China. When clinical bacterial identification was required, two nearly identical wound samples (including wound exudate or wound tissue) were collected, one for standard clinical bacterial identification to give the definitive result and the other immediately sealed in a dedicated testing tube to avoid the loss of volatile metabolites, and tested with GC-IMS as soon as possible. The protocol of this study received ethical approval from the Ethics Committee of Daping Hospital (Approval No.: Yi Yan Lun Shen (2024) NO. 107) and written informed consent was obtained from all participants. The workflow of the research design and data analysis is shown in Figure 1.

### 2.2. GC-IMS Principle and Detection

GC-IMS combines the high separation capability of gas chromatography (GC) with the high sensitivity of ion mobility spectrometry (IMS). First, compounds in a mixed sample are physically separated by gas chromatography. Briefly, VOCs are injected into the chromatographic column and carried by high-purity nitrogen carrier gas. Each compound elutes from the column at a distinct rate based on its differential interactions with the stationary phase coating, yielding the first characteristic parameter: retention time (RT). Then the pre-separated components are tested by IMS. Briefly, each component flowing out of the GC column is ionized (usually using soft ionization methods such as radioactive tritium sources or corona discharge) at atmospheric pressure or near atmospheric pressure and, driven by a weak electric field, these ions pass through a drift tube against a steady gas flow (usually nitrogen). Each ion would take different times to reach the sensor pad on the other side due to differences in their mass, charge, and collision frequency with drift gas molecule (related to the size and shape of each molecule), thus producing the second characteristic parameter: drift time (DT). Thus, each compound yields a unique two-dimensional “fingerprint” in the GC-IMS spectrum, which enables both compound identification and relative quantification [26,27].

GC-IMS analysis was performed using the FlavourSpec^®^ platform (G.A.S, Dortmund, Germany), a hybrid instrument integrating high-resolution gas chromatography with sensitive ion mobility spectrometry. The system featured a wide-bore GC column (mxt-5 15 m × 0.53 mm × 1 μm, RESTEK, Bellefonte, PA, USA). Headspace sampling was automated via an automatic sampling device (G.A.S, Dortmund, Germany), which incorporates programed thermal incubation and shaking to ensure consistent headspace equilibration. High-purity nitrogen carrier gas was generated on-site through an integrated nitrogen generator (G.A.S, Dortmund, Germany), eliminating reliance on external gas cylinders and ensuring uninterrupted, stable flow conditions throughout analysis.

The experimental parameters were as follows: incubation temperature and time were 80 °C and 20 min before sample was injected into the core component, injection volume was 1 mL, and detection time was 30 min. After 20 min of incubation, the sampling syringe extracted the headspace gas 1 mL from the testing bottle containing wound infection sample and injected it into the core component for detection. Temperature settings (drift tube: 45 °C; GC column: 40 °C; injection port: 80 °C) were selected to ensure thermal stability and analyte volatility. Coupled with a steady drift gas flow of 150 mL/min, the system employed a time-dependent gradient in chromatographic column flow to enhance resolution of target volatiles: constantly at 2 mL/min in the first 2 min, linearly increase from 2 mL/min to 10 mL/min in the following 8 min, and linearly increase from 10 mL/min to 80 mL/min in the next 10 min and constantly at 80 mL/min in the last 10 min. To minimize the influence of experimental environment, laboratory temperature and humidity were kept consistent across all experiments. At the same time, to avoid possible carryover effects on the next experiment, we conducted a cleaning procedure to clean the instrument after each experiment.

### 2.3. Data Processing

Due to the fact that positive mode compounds have much more information than negative mode compounds, this study chose positive mode data for biomarker analysis. Each sample would produce a unique two-dimensional GC-IMS spectrum (retention time vs. drift time), in which each dot represents a feature point and the color depth of the dot represents its concentration, as shown in Figure 1. Feature points were manually selected through the dedicated software GC-IMS LAV 2.2.1 (G.A.S, Dortmund, Germany), and the peak height values were exported for subsequent processing by machine learning algorithms. The software has built-in reactive ion peak (RIP) alignment function for drift time offset correction. And, before extracting feature points, all data were aligned with reference to the RIP to eliminate possible drift time offsets. Notably, if the sample was wound exudate and sampled with a cotton swab, a paired blank swab was also detected. Then, feature point values from each sample swab were baseline-corrected by subtracting the values of paired blank swabs. Any resulting negative values were set to the baseline value.

This study employed R for data processing. First, the samples were grouped by presence of infection by any bacteria and by presence of infection by a certain bacterium despite infection status by other bacteria. Then the normalization steps include logarithmic transformation, scaling (Pareto scaling), and batch correction. R function log1p (x) is used for logarithmic transformation to correct the data distribution, where x represents the feature values. Because the data did not have clear batch labels, we used surrogate variable analysis to capture unknown batch effects. If the estimated number of potential batch factors is 0, indicating that the batch effect is not significant, then batch correction would not be performed. Initial feature screening and dimensionality reduction were performed using partial least squares discriminant analysis (PLS-DA) implemented through the ropls package in R [28] and Mann–Whitney U test based on grouping. For subsequent analysis, only those variables demonstrating both significant discriminatory power (variable importance in projection, VIP > 1) and statistical significance (*p*-value < 0.05) were retained. Subsequently, for classification models, support vector machine (SVM), logistic regression (LR), and random forest (RF) were employed, implemented using the e1071 [29], glmnet [30], and randomForest [31] packages, respectively. The model training and evaluation were conducted using 5-fold cross-validation and repeated 10 times to obtain more robust results. The accuracy, sensitivity, specificity, receiver operating characteristic (ROC) curves and area under the curve (AUC) were calculated to evaluate the model. For each classification model, validation runs with sensitivity > 0.6, specificity > 0.6, and AUC > 0.8 were used for feature importance calculation and ranking, and the frequency of features appearing in top 20 features of the valid runs that meet the above conditions was counted. Finally, the most discriminative volatile metabolic biomarkers were identified by combining the high-frequency features of all three models. Additionally, VOCs were annotated through the GC × IMS Library Search 1.0.3 (G.A.S, Dortmund, Germany) by referencing GC × IMS database.

## 3. Results

### 3.1. Data Collection and Preprocessing

To screen for volatile metabolites associated with infection and specific bacterial infections, the samples were categorized by bacterial infection status (infected vs. uninfected) and infection status by a specific bacterium (specific bacterium-positive vs. negative). According to the bacterial identification results from the microbiology laboratory, GC-IMS data collected in this paper are summarized in Table 1. A total of 88 GC-IMS data were collected, including 58 bacterial infections and 30 non-bacterial infections. Among them, *E. coli* infection is the most common, with 14 out of the total. Due to limited sample size, this paper only discusses the classification results for presence of bacterial infection and *E. coli* infection. Identification of other bacterial infections will be analyzed after sufficient samples are collected.

Before extracting feature points, all data were aligned with reference to the reactive ion peak (RIP) to eliminate drift time offsets. A total of 196 features were selected from all data and their height values above area minimum were exported for subsequent analysis, of which 37 features were successfully annotated by comparing with a database. Detailed information of the 37 features is provided in Table S1. As mentioned in the previous research, when the concentration of a certain compound is too high, both monomers and dimers will be produced simultaneously, distributed at different positions on the migration time axis [32,33]. Thus the 37 features belong to 19 compounds, including 5 ketones, 5 alcohols, 6 aldehydes, and 3 esters. After performing logarithmic transformation and Pareto scaling on the feature values, the estimated potential batch factors were 0; therefore, batch correction was not performed.

### 3.2. Differential Metabolite Analysis for Bacterial Infection Status

**Figure 2:**
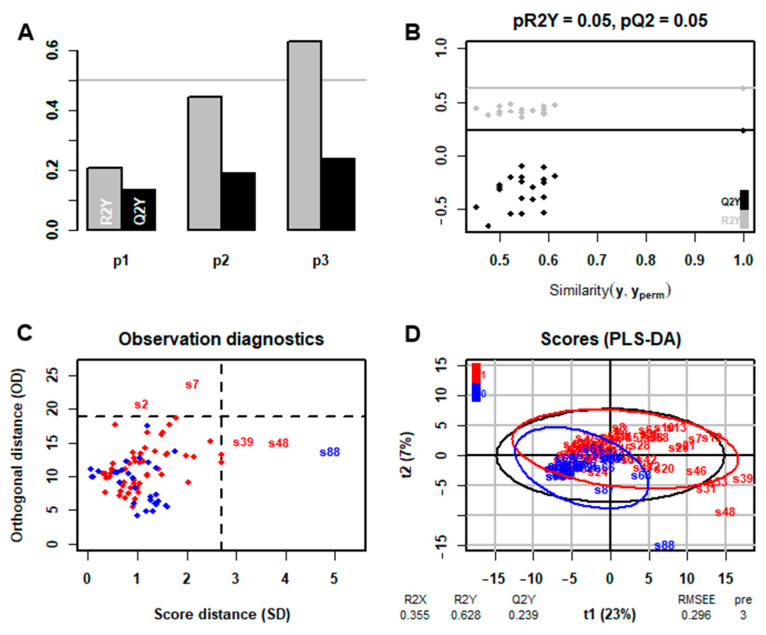
PLS-DA model for identifying infection presence or absence using ropls (R package). (**A**) Orthogonal components that account for the largest proportion of total inertia in the dataset. (**B**) Diagnostic significance assessment: model performance was validated through permutation testing, where the original R2Y and Q2Y values were compared against distributions generated by randomly permuting the response variable (Y) 20 times. (**C**) Outlier detection. (**D**) Scores of PLS-DA, the components number and cumulative R2X, R2Y, and Q2Y are presented below the plot. “1” represents the presence of infection; “0” represents the absence of infection.

The results of PLS-DA by ropls package for identifying the presence of infection are shown in Figure 2. Three orthogonal components captured most of the inertia, and diagnostic significance assessment suggested no overfitting. R2Y of the model is 0.628 and Q2Y is 0.239, indicating acceptable model fit but suboptimal predictive performance. However, without regard to the predictive performance, the VIP scores from PLS-DA were employed in conjunction with non-parametric statistical tests for preliminary screening of feature points to reduce feature dimensionality. Forty-one features with VIP > 1 and *p*-value < 0.05 were selected for the construction of the classification model. Detailed information of the 41 features is provided in Table S2.

**Figure 3:**
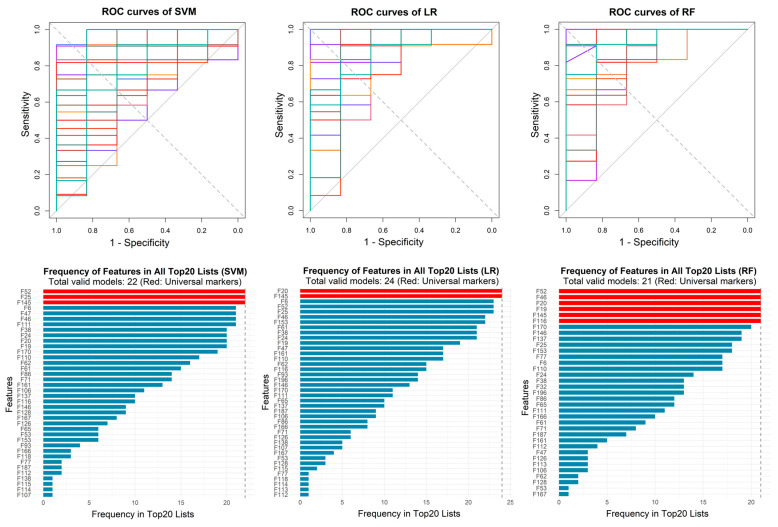
The ROC curves of all valid models used to identify infection presence or absence and the frequency of features appearing in top 20 importance features lists of all valid models. SVM, support vector machine; LR, logistic regression; RF, random forest.

To evaluate the performance of the three classification algorithms (SVM, LR, and RF) for identifying infection presence or absence, the model training was conducted using 5-fold cross-validation, and 10 repeats were performed for each algorithm. In the experiment, the linear kernel was chosen for SVM in order to calculate the feature weights. To prevent overfitting, the logistic regression model included a regularization term and adopted elastic net regression, with a mixed ratio of L1 and L2 regularization of 0.5. The optimal regularization coefficient (lambda) was determined via 3-fold cross-validation for each training run. Then the final model of each training retrained with the optimal lambda and the importance of the features was ranked according to the logistic regression coefficients of each feature. The tree number of the random forest model was set to 500, and the importance of features was ranked according to mean decrease in Gini impurity. Validation runs with sensitivity > 0.6, specificity > 0.6, and AUC > 0.8 were considered valid models. The ROC curves of all valid models and the frequency of features appearing in top 20 importance features lists of all valid models are shown in Figure 3. The classification metrics of all valid models for identifying infection presence or absence are summarized in Table 2, with detailed training results provided in Tables S3–S5.

**Figure 4:**
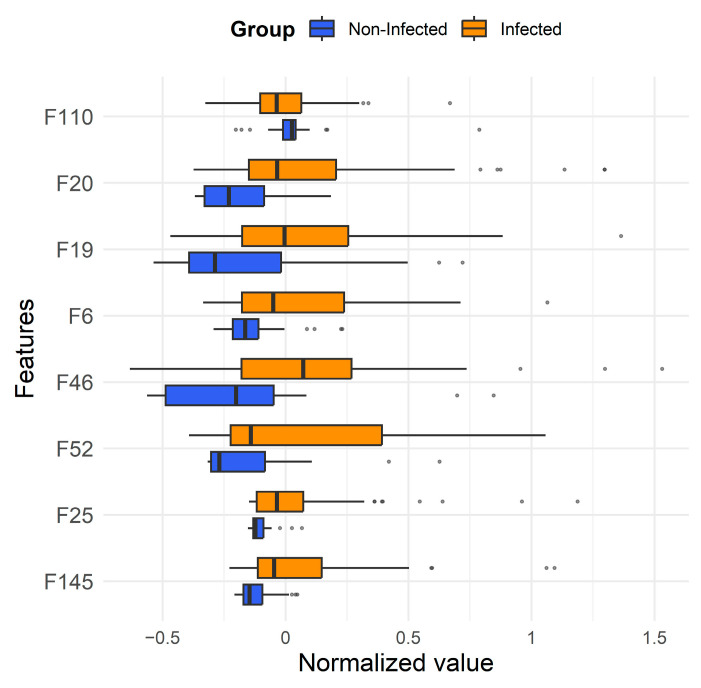
Box plot of characteristic features in Table 3 for identifying infection presence or absence.

Based on the ROC curves and feature importance ranking, eight features that appeared 15 or more times in valid models of all three classification algorithms were considered potential biomarkers, which are listed in Table 3. Among them, F19, F20 and F145 were annotated as 3-methyl-1-butanol monomer, 2-methyl-1-butanol monomer and ethyl hexanoate monomer by referring to a database. The box plots for these features are shown in Figure 4. It can be observed that, except for F110, all other characteristic features showed increased abundance in infected samples.

### 3.3. Differential Metabolite Analysis for the Presence or Absence of E. coli Infection

**Figure 5:**
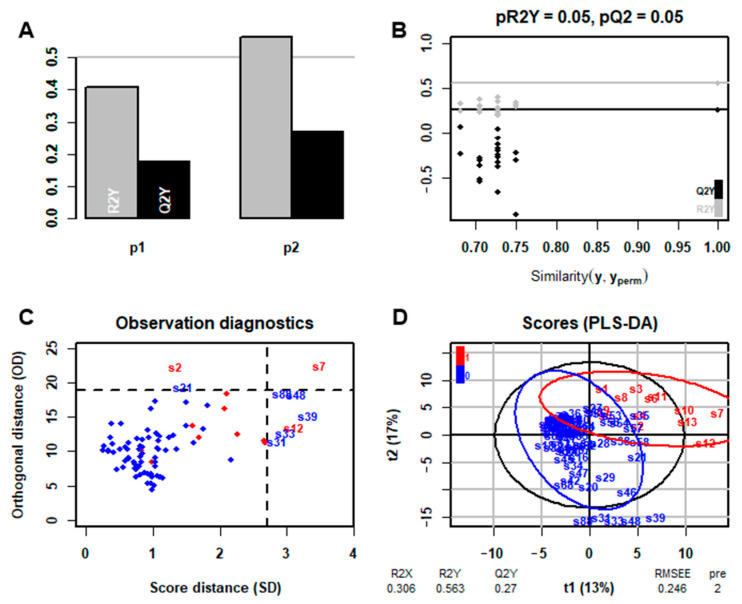
PLS-DA model for identifying *E. coli* infection using ropls (R package). (**A**) Orthogonal components that account for the largest proportion of total inertia in the dataset. (**B**) Diagnostic significance assessment: model performance was validated through permutation testing, where the original R2Y and Q2Y values were compared against distributions generated by randomly permuting the response variable (Y) 20 times. (**C**) Outlier detection. (**D**) Scores of PLS-DA, the components number and cumulative R2X, R2Y, and Q2Y are presented below the plot. “1” represents the presence of *E. coli* infection; “0” represents the absence of *E. coli* infection.

Results of PLS-DA by ropls package for the presence of *E. coli* infection are shown in Figure 5. Two orthogonal components captured most of the inertia, and diagnostic significance assessment suggested no overfitting. R2Y of the model is 0.563 and Q2Y is 0.27, suggesting acceptable model fit but suboptimal predictive performance. Like the PLS-DA model for identifying infection presence or absence, we can still calculate the VIP values for preliminary screening and dimensionality reduction of features. Thirty-two features with VIP > 1 and *p*-value < 0.05 were selected for construction of the classification model. Details of the 32 features are provided in Table S6.

**Figure 6:**
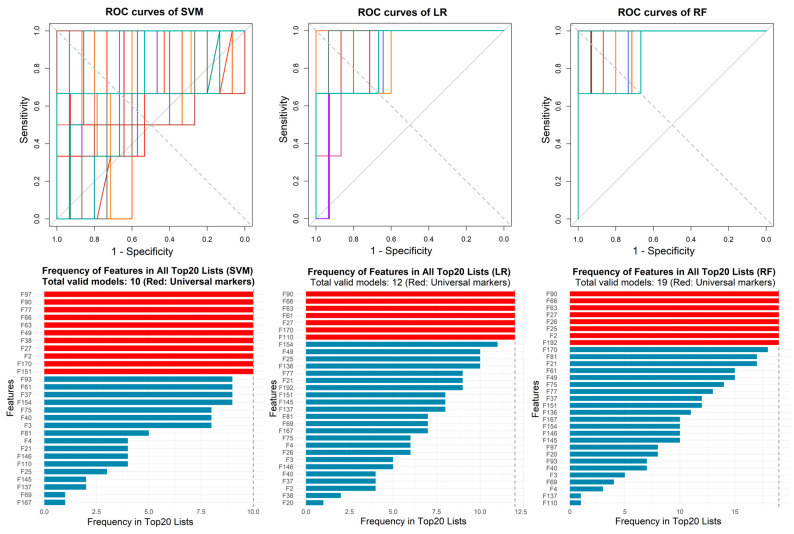
The ROC curves of all valid models used to identify *E. coli* infection presence or absence and the frequency of features appearing in top 20 importance features lists of all valid models. SVM, support vector machine; LR, logistic regression; RF, random forest.

The methods for model training and evaluation are consistent with identifying the presence or absence of infection. The models with sensitivity > 0.6, specificity > 0.6, and AUC > 0.8 were considered valid models. The ROC curves of all valid models and the frequency of features appearing in top 20 importance features lists of all valid models are shown in Figure 6. The classification metrics of all valid models for identifying *E. coli* infection presence or absence are summarized in Table 4, with detailed training results provided in Tables S7–S9.

**Figure 7:**
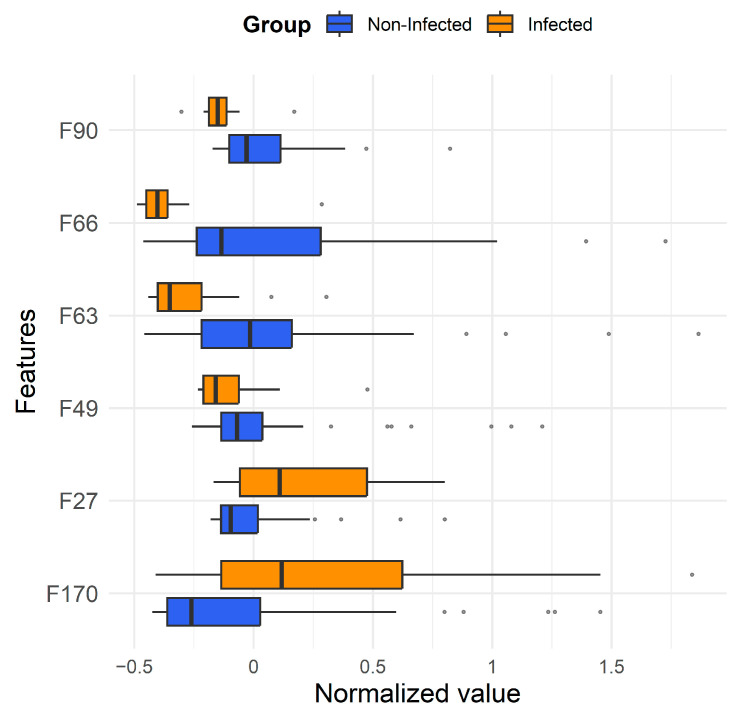
Box plot of characteristic features in Table 5 for identifying *E. coli* infection presence or absence.

Based on the ROC curves and feature importance ranking, six features that appeared 10 or more times in valid models of all three classification algorithms were considered potential biomarkers, which are listed in Table 5. The box plots for these features are shown in Figure 7. It can be seen that, except for F27 and F170, which showed increased abundance in samples with *E. coli* infection, the other features all tend to decrease.

## 4. Discussion

This study extends the detection of bacterial volatile metabolites by GC-IMS from bacterial culture to real complex clinical environments. With the analysis of machine learning algorithms, eight characteristic features that can be used to identify bacterial infection and six characteristic features that can be used to identify *E. coli* infection were selected. This method can rapidly identify bacterial infections, especially *E. coli* infection. The first report that used GC-IMS for direct analysis of wound swabs demonstrated that it could distinguish between infected and non-infected samples with high sensitivity and specificity [27]. This study reproduced the previous study and identified potential volatile biomarkers to detect bacterial infection and *E. coli* infection.

For differential metabolite analysis of infection, 3-methyl-1-butanol, 2-methyl-1-butanol, and ethyl hexanoate were successfully annotated as potential volatile biomarkers. 3-methyl-1-butanol and 2-methyl-1-butanol can be produced by microorganisms through the Ehrlich pathway, where leucine and isoleucine are deaminated into α-keto acids, then decarboxylated and reduced into 3-methyl-1-butanol and 2-methyl-1-butanol, respectively [6,13,34,35,36,37]. Increased concentrations of 3-methyl-1-butanol have been also detected in the headspaces of cultures of *Staphylococcus aureus*, *Pseudomonas aeruginosa*, and *Staphylococcus epidermidis* [38,39,40]. GC-IMS was applied to monitor VOCs released by *Staphylococcus aureus* in refrigerated chicken during the initial phase of storage, and the results suggested 3-methyl-1-butanol as a potential characteristic biomarker of *Staphylococcus aureus* [41]. 3-methyl-1-butanol was also detected in the headspace of the collagen wound biofilm model of *Staphylococcus aureus* [42]. In addition, as *Klebsiella pneumoniae* can also break down leucine to produce 3-methyl-1-butanol via the Ehrlich pathway, 3-methyl-1-butanol was suggested to be a volatile metabolite closely correlated with Enterobacteriaceae bacteria [43,44]. A study used GC-MS to detect VOCs from real wound samples and also detected 3-methyl-1-butanol in the bacterial infection group, speculating that it may be the result of amino acid decomposition [15]. As for 2-methyl-1-butanol, results from a metabolic profiling study on VOCs released by bacteria isolated from pressure ulcer wounds indicated that 2-methyl-1-butanol was elevated in the headspace of *E. coli* and *Proteus mirabilis* cultures, suggesting an enhancement of the Ehrlich pathway [45]. These results demonstrate a correlation between these branched-chain alcohols and wound infection status, resulting from active amino acid catabolism by microorganisms in the wound. Ethyl hexanoate, an important ester compound in wine and spirits, is usually produced by the esterification reaction of hexanoic acid and ethanol during the later stages of microbial fermentation [46]. The elevation of these branched-chain alcohols and ester metabolites directly reflects active microbial amino acid catabolism and fermentation in the wound bed, highlighting their potential as volatile metabolic markers of active wound infection.

For differential metabolite analysis of *E. coli* infection, six characteristic features were selected through consensus among three machine learning algorithms. All of these features showed stable differences between *E. coli* infected and non-*E. coli* infected wound samples and served as core features of the classification models. However, none of them were annotated through the current GC-IMS commercial database. Yet, it should be emphasized that the lack of clear structural annotations will not reduce the potential clinical diagnostic value of these features. Thus, they can be used as specific volatile “fingerprint features” for rapid identification of *E. coli* infections in wounds.

The results in this study showed that, despite the complex sample backgrounds and limited sample size, the proposed method still exhibits good classification performance. The key lies in effectively capturing the characteristic VOCs “fingerprint” generated by bacterial metabolic activity. This study provides a promising new strategy for rapid bedside diagnosis of wound infections.

Despite the promising results, this study has several limitations. First, the sample size (n = 88) is relatively small for establishing robust diagnostic models. In particular, models for *Staphylococcus aureus* and *Pseudomonas aeruginosa* were not possible due to insufficient sample size (n = 6 each). Wound samples will be continuously collected to build more robust classification models and extend coverage to other types of wound-infecting bacteria. Second, most clinical wound infections are caused by mixed microbial communities, yet the current classification model (“presence of specific bacteria”) simplified such complexity. Future studies should systematically include single-bacterial and mixed-infection samples to analyze the competitive or additive effects of volatile metabolites profiles in mixed-infection states. Third, the specific chemical structures of many high-contribution features could not be identified, although this seems a common challenge in the entire field of GC-IMS applications. A more comprehensive IMS standard spectral database that includes characteristic bacterial volatile metabolites would greatly advance diagnostic applications of this technique. Finally, after getting VOC profile with GC-IMS, existing feature point extraction methods mainly rely on manual selection in the system’s software, which is highly subjective. Future work will incorporate automated feature extraction algorithms to eliminate the inter-operator variability and omissions associated with manual feature selection and further improve the stability of the diagnostic model.

## 5. Conclusions

The results of this study confirm that, by detecting bacterial volatile metabolites with GC-IMS, followed by analysis with statistical and machine learning algorithms, bacterial infection status in clinical wound samples can be rapidly and accurately diagnosed. This method is faster than traditional biological methods, demonstrating the potential for early diagnosis and bedside applications in clinical settings. The key finding of this study is that, by constructing a classification model and screening differentially expressed volatile metabolites, GC-IMS data can be used to train a diagnostic model with high sensitivity and specificity. These findings provide critical experimental evidence and a technical framework for the development of a non-invasive, point-of-care diagnostic device for wound infections, which has the potential to reduce empirical antibiotic use and improve patient outcomes through timely targeted treatment.

## Figures and Tables

**Table 1:** Quantity of GC-IMS data collection.

Groups	With	Without
With or without bacterial infection	58	30
With or without *Escherichia coli* infection	14	74
With or without *Staphylococcus aureus* infection	6	82
With or without *Pseudomonas aeruginosa* infection	6	82

**Table 2:** Classification metrics of all valid models for identifying infection presence or absence.

Metrics	SVM		LR		RF	
	Mean	95% CI	Mean	95% CI	Mean	95% CI
Accuracy (%)	81.55	78.85–84.25	83.48	80.64–86.32	85.19	82.28–88.09
Sensitivity (%)	81.44	77.82–85.06	85.35	81.42–89.29	90.15	86.15–94.16
Specificity (%)	81.82	77.31–86.33	79.86	75.23–84.49	75.40	69.71–81.08
AUC	0.90	0.88–0.92	0.89	0.87–0.92	0.91	0.88–0.95

**Table 3:** Features that appear 15 or more times in valid models of all three classification algorithms to identify infection presence or absence.

Feature Index	RT (s)	DT (RIPrel)	*p*-Value	VIP	Annotation	Formula
F110	598.605	1.205	4.15 × 10^−2^	1.436362		
F20	218.295	1.233	1.92 × 10^−5^	1.240365	2-methyl-1-butanol monomer	C_5_H_12_O
F19	202.020	1.252	1.09 × 10^−3^	1.40215	3-methyl-1-butanol monomer	C_5_H_12_O
F6	122.745	1.198	7.85 × 10^−3^	1.335586		
F46	576.870	1.151	4.22 × 10^−4^	1.662901		
F52	664.860	1.262	1.77 × 10^−4^	1.344279		
F25	218.820	1.404	2.51 × 10^−4^	1.16806		
F145	609.210	1.347	9.47 × 10^−6^	1.13919	ethyl hexanoate monomer	C_8_H_16_O_2_

**Table 4:** Classification metrics of all valid models for identifying *E. coli* infection.

Metrics	SVM		LR		RF	
	Mean	95% CI	Mean	95% CI	Mean	95% CI
Accuracy (%)	90.14	86.73–93.56	83.48	80.64–86.32	95.56	94.12–97.01
Sensitivity (%)	72.22	63.98–80.47	85.35	81.42–89.29	77.19	69.52–84.87
Specificity (%)	93.81	90.44–97.18	79.86	75.23–84.49	99.30	98.29–100.00
AUC	0.92	0.89–0.96	0.89	0.87–0.92	0.97	0.95–0.99

**Table 5:** Features that appear 10 or more times in valid models of all three classification algorithms to identify *E. coli* infection presence or absence.

Feature Index	RT (s)	DT (RIPrel)	*p*-Value	VIP
F90	975.870	1.819	5.10 × 10^−5^	1.797587
F66	607.950	1.689	1.43 × 10^−6^	1.634912
F63	285.915	1.483	3.41 × 10^−4^	1.41761
F49	578.445	1.679	3.63 × 10^−2^	1.016113
F27	220.920	1.465	2.49 × 10^−3^	1.913331
F170	153.300	1.405	5.97 × 10^−3^	1.562065

## Data Availability

Data supporting this study are available from the corresponding author upon reasonable request.

## Supplementary Materials

The following supporting information can be downloaded at: https://www.mdpi.com/article/10.3390/metabo16040272/s1, Table S1: All VOCs annotated successfully; Table S2: Forty-one feature points selected for identifying infection status through screening with VIP > 1 and *p*-value < 0.05.; Tables S3–S5: Detailed training results of SVM, LR and RF for identifying infection presence or absence.; Table S6: Thirty-two feature points selected for identifying *E. coli* infection through screening with VIP > 1 and *p*-value < 0.05; Tables S7–S9: Detailed training results of SVM, LR and RF for identifying *E. coli* infection presence or absence.

## Abbreviations

The following abbreviations are used in this manuscript:

VOCs	Volatile organic compounds
GC-IMS	Gas chromatography-ion mobility spectrometry
PLS-DA	Partial least squares discriminant analysis
VIP	Variable importance in projection
SVM	Support vector machine
LR	Logistic regression
RF	Random Forest
*E. coli*	*Escherichia coli*
AUC	Area under the curve
GC-MS	Gas chromatography-mass spectrometry
RIP	Reactive ion peak
ROC	Receiver operating characteristic
RT	Retention time
DT	Drift time
