# Supplementary material for: Untargeted GC-IMS Metabolomics of Wound Headspace for Bacterial Infection Biomarker Discovery
Source: Metabolites. 2026 Apr 17;16(4):272. doi: 10.3390/metabo16040272 (PMC13117574; doi:10.3390/metabo16040272)
Supplement: Supplementary file 1 [file metabolites-16-00272-s001.zip › metabolites-4252594-supplementary.pdf]

Table S1. All VOCs annotated successfully.

Index	Annotated compound	CAS#	Formula	Rt(s)	Dt(RIPrel)
F2	2-butanone monomer	78-93-3	C_4H_8O	135.345	1.064
F3	butanal monomer	123-72-8	C_4H_8O	153.720	1.112
F9	butanal dimer	123-72-8	C_4H_8O	129.570	1.289
F14	ethyl acetate dimer	141-78-6	$C_4H_8O_2$	140.595	1.337
F18	2-pentanone monomer	107-87-9	$C_5H_{10}O$	195.195	1.120
F19	3-methyl-1-butanol monomer	123-51-3	$C_5H_{12}O$	202.020	1.252
F20	2-methyl-1-butanol monomer	137-32-6	$C_5H_{12}O$	218.295	1.233
F23	1-pentanol monomer	71-41-0	$C_5H_{12}O$	244.020	1.255
F26	3-methyl-1-butanol dimer	123-51-3	$C_5H_{12}O$	210.945	1.500
F28	hexanal monomer	66-25-1	$C_6H_{12}O$	274.470	1.294
F30	1-pentanol dimer	71-41-0	$C_5H_{12}O$	240.870	1.522
F31	hexanal dimer	66-25-1	$C_6H_{12}O$	271.845	1.562
F34	2-heptanone monomer	110-43-0	$C_7H_{14}O$	382.830	1.257
F35	cyclohexanone monomer	108-94-1	$C_6H_{10}O$	419.580	1.152
F36	cyclohexanone dimer	108-94-1	$C_6H_{10}O$	410.655	1.449
F37	2-heptanone dimer	110-43-0	$C_7H_{14}O$	380.205	1.625
F38	1-hexanol monomer	111-27-3	$C_6H_{14}O$	367.080	1.326
F39	1-hexanol dimer	111-27-3	$C_6H_{14}O$	360.255	1.653
F41	benzaldehyde monomer	100-52-7	C_7H_6O	512.505	1.147
F42	(E)-2-heptenal monomer	18829-55-5	$C_7H_{12}O$	520.380	1.251
F43	benzaldehyde dimer	100-52-7	C_7H_6O	509.355	1.461
F45	(E)-2-heptenal dimer	18829-55-5	$C_7H_{12}O$	502.005	1.663
F51	ethyl hexanoate dimer	123-66-0	$C_8H_{16}O_2$	612.045	1.818
F53	phenylacetaldehyde monomer	122-78-1	C_8H_8O	689.010	1.264
F60	2-pentenal monomer	764-39-6	C_5H_8O	224.700	1.104
F69	ethyl acetate monomer	141-78-6	$C_4H_8O_2$	140.595	1.095
F72	propyl butanoate dimer	105-66-8	$C_7H_{14}O_2$	404.145	1.691
F73	2-butanone dimer	78-93-3	C_4H_8O	130.095	1.271
F81	propyl butanoate monomer	105-66-8	$C_7H_{14}O_2$	405.720	1.268
F106	2-pentenal dimer	764-39-6	C_5H_8O	225.540	1.361
F116	acetone	67-64-1	C_3H_6O	97.860	1.094
F117	ethanol dimer	64-17-5	C_2H_6O	97.860	1.124
F121	ethanol monomer	64-17-5	C_2H_6O	90.825	1.046
F141	phenylacetaldehyde dimer	122-78-1	C_8H_8O	683.760	1.542
F145	ethyl hexanoate monomer	123-66-0	$C_8H_{16}O_2$	609.210	1.347
F169	2-pentanone dimer	107-87-9	$C_5H_{10}O$	184.590	1.394
F191	2-methyl-1-butanol dimer	137-32-6	$C_5H_{12}O$	226.380	1.486

Table S2. Forty-one feature points selected for identifying infection status through screening with VIP > 1 and p-value < 0.05.

**Index**	**p-value**	**VIP**
**F106**	1.26×10^{-3}	1.300753
**F107**	1.60×10^{-2}	1.180802
**F110**	4.15×10^{-2}	1.436362
**F111**	8.74×10^{-4}	1.006952
**F112**	4.46×10^{-3}	1.052308
**F113**	5.82×10^{-3}	1.392451
**F114**	2.61×10^{-2}	1.343721
**F115**	6.35×10^{-3}	1.048459
**F116**	5.41×10^{-3}	1.986132
**F118**	3.50×10^{-3}	1.172037
**F126**	1.92×10^{-2}	1.005304
**F128**	3.72×10^{-3}	1.282032
**F137**	7.34×10^{-3}	1.312438
**F138**	3.07×10^{-2}	1.08222
**F145**	9.47×10^{-6}	1.13919
**F146**	1.21×10^{-4}	1.105855
**F153**	4.33×10^{-2}	1.495017
**F161**	1.77×10^{-3}	1.229759
**F166**	1.07×10^{-2}	1.046991
**F167**	2.36×10^{-2}	1.142969
**F170**	2.15×10^{-3}	1.048004
**F187**	4.39×10^{-3}	1.371489
**F19**	1.09×10^{-3}	1.40215
**F196**	4.34×10^{-3}	1.280396
**F20**	1.92×10^{-5}	1.240365
**F24**	3.14×10^{-2}	1.209172
**F25**	2.51×10^{-4}	1.16806
**F32**	1.78×10^{-3}	1.106991
**F38**	6.61×10^{-3}	1.145331
**F46**	4.22×10^{-4}	1.662901
**F47**	2.25×10^{-3}	1.134927
**F52**	1.77×10^{-4}	1.344279
**F53**	1.08×10^{-2}	1.034321
**F6**	7.85×10^{-3}	1.335586
**F61**	2.84×10^{-2}	1.264491
**F62**	1.04×10^{-3}	1.08537

**F65**	9.39×10^{-3}	1.049984
**F71**	6.02×10^{-3}	1.02402
**F77**	2.21×10^{-4}	1.247714
**F86**	1.57×10^{-3}	1.132297
**F93**	3.16×10^{-2}	1.168498

Table S3. Detailed training results of SVM for identifying infection presence or absence.

**Experiment Index**	**Fold**	**Accuracy**	**Sensitivity**	**Specificity**	**AUC**	**AUC_CI_low**	**AUC_CI_high**	**Is_Valid**
1	1	0.9444	0.9167	1.0000	0.9722	0.9078	1.0000	TRUE
1	3	0.8333	0.8333	0.8333	0.9167	0.7902	1.0000	TRUE
1	4	0.7647	0.8182	0.6667	0.8182	0.5224	1.0000	TRUE
2	2	0.7222	0.6667	0.8333	0.8333	0.6409	1.0000	TRUE
2	4	0.8235	0.8182	0.8333	0.9242	0.7997	1.0000	TRUE
2	5	0.8333	0.7500	1.0000	0.8333	0.6355	1.0000	TRUE
3	2	0.8235	0.8182	0.8333	0.9091	0.7630	1.0000	TRUE
3	3	0.8824	0.9091	0.8333	0.9242	0.7671	1.0000	TRUE
3	4	0.7778	0.8333	0.6667	0.8611	0.6586	1.0000	TRUE
4	1	0.9412	0.9091	1.0000	0.9848	0.9429	1.0000	TRUE
4	4	0.8333	0.8333	0.8333	0.9722	0.9066	1.0000	TRUE
5	2	0.8235	0.8182	0.8333	0.9242	0.7983	1.0000	TRUE
5	3	0.8889	0.9167	0.8333	0.9028	0.7480	1.0000	TRUE
5	5	0.7059	0.6364	0.8333	0.8485	0.6524	1.0000	TRUE
6	3	0.8235	0.9091	0.6667	0.8333	0.6033	1.0000	TRUE
6	5	0.8235	0.9091	0.6667	0.8939	0.7302	1.0000	TRUE
7	4	0.7778	0.7500	0.8333	0.8194	0.5220	1.0000	TRUE
8	4	0.7778	0.7500	0.8333	0.9306	0.7860	1.0000	TRUE
9	2	0.7647	0.7273	0.8333	0.9242	0.7990	1.0000	TRUE
9	4	0.7778	0.8333	0.6667	0.8750	0.6941	1.0000	TRUE
9	5	0.7647	0.7273	0.8333	0.9242	0.7895	1.0000	TRUE
10	4	0.8333	0.8333	0.8333	0.9444	0.8261	1.0000	TRUE
1	2	0.7059	0.8182	0.5000	0.6667	0.3868	0.9465	FALSE
1	5	0.5556	0.5833	0.5000	0.5972	0.2862	0.9082	FALSE
2	1	0.8235	1.0000	0.5000	0.9091	0.7654	1.0000	FALSE
2	3	0.5000	0.5000	0.5000	0.6667	0.4012	0.9322	FALSE
3	1	0.7222	0.8333	0.5000	0.6667	0.4015	0.9319	FALSE
3	5	0.7778	0.8333	0.6667	0.7917	0.5492	1.0000	FALSE
4	2	0.7647	0.9091	0.5000	0.7576	0.4645	1.0000	FALSE
4	3	0.6667	0.7500	0.5000	0.7083	0.4108	1.0000	FALSE
4	5	0.7222	0.8333	0.5000	0.7222	0.4263	1.0000	FALSE
5	1	0.7778	0.8333	0.6667	0.7639	0.4824	1.0000	FALSE
5	4	0.7778	0.8333	0.6667	0.7361	0.3999	1.0000	FALSE

6	1	0.6667	0.5833	0.8333	0.7639	0.5378	0.9900	FALSE
6	2	0.7222	0.8333	0.5000	0.7639	0.5073	1.0000	FALSE
6	4	0.8333	0.8333	0.8333	0.7778	0.4955	1.0000	FALSE
7	1	0.6667	0.5833	0.8333	0.7361	0.4663	1.0000	FALSE
7	2	0.6667	0.7500	0.5000	0.8472	0.6586	1.0000	FALSE
7	3	0.6471	0.7273	0.5000	0.6515	0.3633	0.9397	FALSE
7	5	0.6471	0.9091	0.1667	0.6970	0.4330	0.9609	FALSE
8	1	0.7059	0.8182	0.5000	0.9091	0.7654	1.0000	FALSE
8	2	0.8235	0.9091	0.6667	0.6818	0.3557	1.0000	FALSE
8	3	0.6667	0.6667	0.6667	0.7917	0.5633	1.0000	FALSE
8	5	0.6111	0.5833	0.6667	0.6944	0.4082	0.9807	FALSE
9	1	0.8333	0.9167	0.6667	0.7778	0.5204	1.0000	FALSE
9	3	0.7222	0.7500	0.6667	0.6944	0.4330	0.9559	FALSE
10	1	0.7647	0.9091	0.5000	0.7727	0.5204	1.0000	FALSE
10	2	0.6471	0.5455	0.8333	0.8788	0.6368	1.0000	FALSE
10	3	0.7778	0.9167	0.5000	0.7639	0.4654	1.0000	FALSE
10	5	0.6667	0.7500	0.5000	0.7361	0.4569	1.0000	FALSE

Table S4. Detailed training results of LR for identifying infection presence or absence.

Experiment Index	Fold	Accuracy	Sensitivity	Specificity	AUC	AUC_CI_low	AUC_CI_high	Is_Valid
1	1	1.0000	1.0000	1.0000	1.0000	1.0000	1.0000	TRUE
1	3	0.9444	1.0000	0.8333	1.0000	1.0000	1.0000	TRUE
2	2	0.8889	1.0000	0.6667	0.8333	0.6041	1.0000	TRUE
2	4	0.7647	0.8182	0.6667	0.8939	0.7408	1.0000	TRUE
2	5	0.9444	0.9167	1.0000	0.9861	0.9476	1.0000	TRUE
3	1	0.7778	0.8333	0.6667	0.8056	0.5761	1.0000	TRUE
3	2	0.8235	0.9091	0.6667	0.9394	0.8299	1.0000	TRUE
3	3	0.8824	0.9091	0.8333	0.9394	0.8106	1.0000	TRUE
3	4	0.8889	0.9167	0.8333	0.8611	0.6374	1.0000	TRUE
3	5	0.8333	0.9167	0.6667	0.8333	0.6045	1.0000	TRUE
5	3	0.8889	0.9167	0.8333	0.9028	0.7480	1.0000	TRUE
5	5	0.7059	0.6364	0.8333	0.8333	0.6293	1.0000	TRUE
6	1	0.8333	0.7500	1.0000	0.9722	0.9066	1.0000	TRUE
6	4	0.8333	0.8333	0.8333	0.8056	0.5627	1.0000	TRUE
7	1	0.8333	0.8333	0.8333	0.9167	0.7689	1.0000	TRUE
7	4	0.7778	0.7500	0.8333	0.8472	0.5465	1.0000	TRUE
8	3	0.7778	0.7500	0.8333	0.8194	0.6136	1.0000	TRUE
8	4	0.7778	0.7500	0.8333	0.8750	0.6901	1.0000	TRUE
9	2	0.8235	0.9091	0.6667	0.8788	0.7113	1.0000	TRUE
9	4	0.8333	0.9167	0.6667	0.9028	0.7475	1.0000	TRUE
9	5	0.8235	0.8182	0.8333	0.9242	0.7671	1.0000	TRUE

10	2	0.8235	0.8182	0.8333	0.8485	0.5802	1.0000	TRUE
10	4	0.7778	0.7500	0.8333	0.9028	0.7605	1.0000	TRUE
10	5	0.7778	0.8333	0.6667	0.8611	0.6837	1.0000	TRUE
1	2	0.5882	0.7273	0.3333	0.6970	0.4354	0.9586	FALSE
1	4	0.7059	0.9091	0.3333	0.7879	0.5606	1.0000	FALSE
1	5	0.6667	0.6667	0.6667	0.7222	0.4083	1.0000	FALSE
2	1	0.7059	0.9091	0.3333	0.8333	0.6319	1.0000	FALSE
2	3	0.6667	0.6667	0.6667	0.6667	0.4024	0.9309	FALSE
4	1	0.8235	1.0000	0.5000	0.7879	0.5441	1.0000	FALSE
4	2	0.7059	0.8182	0.5000	0.7121	0.4536	0.9707	FALSE
4	3	0.6667	0.7500	0.5000	0.7361	0.4703	1.0000	FALSE
4	4	0.7778	0.9167	0.5000	0.9444	0.8302	1.0000	FALSE
4	5	0.7222	0.8333	0.5000	0.7361	0.4464	1.0000	FALSE
5	1	0.7222	0.8333	0.5000	0.8889	0.7365	1.0000	FALSE
5	2	0.7647	0.9091	0.5000	0.8030	0.5753	1.0000	FALSE
5	4	0.7778	0.8333	0.6667	0.7222	0.3647	1.0000	FALSE
6	2	0.6111	0.6667	0.5000	0.7222	0.4397	1.0000	FALSE
6	3	0.7647	0.9091	0.5000	0.7727	0.4945	1.0000	FALSE
6	5	0.7647	0.9091	0.5000	0.8788	0.6929	1.0000	FALSE
7	2	0.7222	0.8333	0.5000	0.8194	0.6136	1.0000	FALSE
7	3	0.6471	0.7273	0.5000	0.6970	0.4232	0.9708	FALSE
7	5	0.6471	0.9091	0.1667	0.6667	0.3875	0.9459	FALSE
8	1	0.7647	0.9091	0.5000	0.8788	0.7129	1.0000	FALSE
8	2	0.8235	0.9091	0.6667	0.7121	0.4047	1.0000	FALSE
8	5	0.7222	0.7500	0.6667	0.6667	0.3315	1.0000	FALSE
9	1	0.7778	0.8333	0.6667	0.7778	0.5204	1.0000	FALSE
9	3	0.7778	0.9167	0.5000	0.7500	0.4488	1.0000	FALSE
10	1	0.7059	0.9091	0.3333	0.7424	0.4926	0.9923	FALSE
10	3	0.7778	0.9167	0.5000	0.8333	0.6209	1.0000	FALSE

Table S5. Detailed training results of RF for identifying infection presence or absence.

Experiment Index	Fold	Accuracy	Sensitivity	Specificity	AUC	AUC_CI_low	AUC_CI_high	Is_Valid
1	1	0.7778	0.7500	0.8333	0.9444	0.8430	1.0000	TRUE
1	3	0.9444	1.0000	0.8333	1.0000	1.0000	1.0000	TRUE
2	4	0.9412	0.9091	1.0000	0.9848	0.9429	1.0000	TRUE
2	5	1.0000	1.0000	1.0000	1.0000	1.0000	1.0000	TRUE
3	3	0.8235	0.7273	1.0000	0.9773	0.9225	1.0000	TRUE
3	5	0.8333	0.9167	0.6667	0.8333	0.6041	1.0000	TRUE
4	3	0.8889	1.0000	0.6667	0.8056	0.5251	1.0000	TRUE
4	4	0.8333	0.9167	0.6667	0.9722	0.9078	1.0000	TRUE
4	5	0.7778	0.8333	0.6667	0.8333	0.6041	1.0000	TRUE
5	1	0.7778	0.8333	0.6667	0.8333	0.6409	1.0000	TRUE

5	5	0.8824	1.0000	0.6667	0.9242	0.7983	1.0000	TRUE
6	1	0.8889	1.0000	0.6667	0.9306	0.8065	1.0000	TRUE
6	4	0.8889	1.0000	0.6667	0.9861	0.9476	1.0000	TRUE
6	5	0.7647	0.8182	0.6667	0.8636	0.6860	1.0000	TRUE
7	1	0.8889	1.0000	0.6667	0.9583	0.8662	1.0000	TRUE
7	3	0.8235	0.9091	0.6667	0.8333	0.5895	1.0000	TRUE
7	4	0.8333	0.8333	0.8333	0.9306	0.7860	1.0000	TRUE
8	1	0.7647	0.8182	0.6667	0.8636	0.6796	1.0000	TRUE
8	3	0.8333	0.8333	0.8333	0.8333	0.6045	1.0000	TRUE
10	4	0.8333	0.9167	0.6667	0.9722	0.9078	1.0000	TRUE
10	5	0.8889	0.9167	0.8333	0.9306	0.8153	1.0000	TRUE
1	2	0.7647	1.0000	0.3333	0.8788	0.7026	1.0000	FALSE
1	4	0.6471	0.9091	0.1667	0.7424	0.4880	0.9968	FALSE
1	5	0.7778	0.8333	0.6667	0.7222	0.4391	1.0000	FALSE
2	1	0.7647	1.0000	0.3333	0.8030	0.5868	1.0000	FALSE
2	2	0.7778	0.9167	0.5000	0.8472	0.6535	1.0000	FALSE
2	3	0.5000	0.6667	0.1667	0.5417	0.2421	0.8412	FALSE
3	1	0.7778	1.0000	0.3333	0.9028	0.7605	1.0000	FALSE
3	2	0.7059	0.9091	0.3333	0.8030	0.5868	1.0000	FALSE
3	4	0.7222	0.7500	0.6667	0.7917	0.5135	1.0000	FALSE
4	1	0.7647	0.9091	0.5000	0.7273	0.4415	1.0000	FALSE
4	2	0.7059	0.9091	0.3333	0.6515	0.3590	0.9440	FALSE
5	2	0.7647	0.9091	0.5000	0.8182	0.5983	1.0000	FALSE
5	3	0.7778	1.0000	0.3333	0.8194	0.6183	1.0000	FALSE
5	4	0.7222	0.7500	0.6667	0.7778	0.4514	1.0000	FALSE
6	2	0.7222	0.8333	0.5000	0.7083	0.4141	1.0000	FALSE
6	3	0.7647	0.9091	0.5000	0.7879	0.5103	1.0000	FALSE
7	2	0.7778	0.9167	0.5000	0.7917	0.5492	1.0000	FALSE
7	5	0.6471	0.9091	0.1667	0.6818	0.4123	0.9514	FALSE
8	2	0.7647	0.9091	0.5000	0.7652	0.4901	1.0000	FALSE
8	4	0.7222	0.9167	0.3333	0.8125	0.5792	1.0000	FALSE
8	5	0.7778	0.9167	0.5000	0.7847	0.5490	1.0000	FALSE
9	1	0.8333	1.0000	0.5000	0.8681	0.6651	1.0000	FALSE
9	2	0.7647	0.9091	0.5000	0.7879	0.5147	1.0000	FALSE
9	3	0.8333	1.0000	0.5000	0.9444	0.8261	1.0000	FALSE
9	4	0.8333	0.9167	0.6667	0.7986	0.5320	1.0000	FALSE
9	5	0.7059	0.8182	0.5000	0.8182	0.6040	1.0000	FALSE
10	1	0.8235	1.0000	0.5000	0.7652	0.5066	1.0000	FALSE
10	2	0.7059	0.8182	0.5000	0.7652	0.4766	1.0000	FALSE
10	3	0.6111	0.8333	0.1667	0.6111	0.3338	0.8884	FALSE

Table S6. Thirty-two feature points selected for identifying *E. coli* infection through screening

with VIP > 1 and p-value < 0.05.

Index	p-value	VIP
**F110**	9.42×10^{-3}	1.155989
**F136**	1.76×10^{-2}	1.2071
**F137**	2.46×10^{-2}	1.113374
**F145**	1.93×10^{-2}	1.959052
**F146**	2.28×10^{-2}	1.732652
**F151**	4.16×10^{-2}	1.04573
**F154**	4.61×10^{-2}	1.127666
**F167**	1.71×10^{-2}	1.630647
**F170**	5.97×10^{-3}	1.562065
**F192**	3.56×10^{-2}	2.198496
**F2**	1.35×10^{-3}	1.321505
**F20**	9.29×10^{-3}	1.59222
**F21**	3.04×10^{-4}	1.951955
**F25**	3.01×10^{-4}	1.980838
**F26**	1.79×10^{-2}	1.616639
**F27**	2.49×10^{-3}	1.913331
**F3**	4.58×10^{-3}	1.318868
**F37**	1.42×10^{-2}	1.371437
**F38**	1.61×10^{-2}	1.00872
**F4**	2.50×10^{-2}	1.078428
**F40**	1.83×10^{-3}	1.017173
**F49**	3.63×10^{-2}	1.016113
**F61**	4.84×10^{-2}	1.625727
**F63**	3.41×10^{-4}	1.41761
**F66**	1.43×10^{-6}	1.634912
**F69**	1.25×10^{-2}	1.357258
**F75**	1.58×10^{-2}	1.247793
**F77**	1.04×10^{-2}	2.073011
**F81**	1.39×10^{-2}	1.53982
**F90**	5.10×10^{-5}	1.797587
**F93**	1.26×10^{-2}	1.082414
**F97**	3.94×10^{-2}	1.218321

Table S7. Detailed training results of SVM for identifying *E. coli* infection presence or absence.

Experiment Index	Fold	Accuracy	Sensitivity	Specificity	AUC	AUC_CI_low	AUC_CI_high	Is_Valid
1	2	0.7778	0.6667	0.8000	0.8444	0.5274	1.0000	TRUE
1	3	0.7647	0.6667	0.7857	0.8095	0.5576	1.0000	TRUE

2	1	0.8235	1.0000	0.8000	0.9333	0.8027	1.0000	TRUE
3	2	0.9375	1.0000	0.9286	1.0000	1.0000	1.0000	TRUE
4	3	0.8889	1.0000	0.8667	0.9556	0.8501	1.0000	TRUE
5	3	0.8333	0.6667	0.8667	0.9111	0.7584	1.0000	TRUE
6	4	0.8333	0.6667	0.8667	0.9111	0.7584	1.0000	TRUE
8	5	0.8889	1.0000	0.8667	1.0000	1.0000	1.0000	TRUE
9	3	0.8889	1.0000	0.8667	1.0000	1.0000	1.0000	TRUE
10	1	0.8889	0.6667	0.9333	0.8889	0.6561	1.0000	TRUE
1	1	0.7778	0.6667	0.8000	0.7556	0.3282	1.0000	FALSE
1	4	0.9412	0.5000	1.0000	0.7000	0.0982	1.0000	FALSE
1	5	0.8333	0.3333	0.9333	0.7333	0.3638	1.0000	FALSE
2	2	0.8824	0.3333	1.0000	0.7143	0.3818	1.0000	FALSE
2	3	0.7222	0.6667	0.7333	0.7333	0.4881	0.9786	FALSE
2	4	0.7778	0.3333	0.8667	0.6889	0.3718	1.0000	FALSE
2	5	0.8333	0.3333	0.9333	0.6667	0.1325	1.0000	FALSE
3	1	0.8333	0.0000	1.0000	0.6000	0.1908	1.0000	FALSE
3	3	0.8333	0.6667	0.8667	0.6667	0.1904	1.0000	FALSE
3	4	0.7778	0.3333	0.8667	0.6889	0.2860	1.0000	FALSE
3	5	0.7222	0.6667	0.7333	0.7333	0.2060	1.0000	FALSE
4	1	0.8824	0.5000	0.9333	0.8667	0.6676	1.0000	FALSE
4	2	0.7778	0.0000	0.9333	0.6889	0.4475	0.9303	FALSE
4	4	0.8333	0.3333	0.9333	0.6444	0.1685	1.0000	FALSE
4	5	0.7647	0.3333	0.8571	0.6905	0.2566	1.0000	FALSE
5	1	0.7222	0.3333	0.8000	0.6444	0.1213	1.0000	FALSE
5	2	0.8235	0.5000	0.8667	0.9333	0.7752	1.0000	FALSE
5	4	0.7059	0.0000	0.8571	0.5714	0.2213	0.9216	FALSE
5	5	0.7222	0.3333	0.8000	0.7778	0.5018	1.0000	FALSE
6	1	0.8889	0.3333	1.0000	0.7778	0.5018	1.0000	FALSE
6	2	0.7778	0.3333	0.8667	0.7556	0.4638	1.0000	FALSE
6	3	0.7647	0.3333	0.8571	0.8095	0.5576	1.0000	FALSE
6	5	0.6471	0.0000	0.7333	0.4667	0.1227	0.8106	FALSE
7	1	0.8333	0.3333	0.9333	0.6667	0.0740	1.0000	FALSE
7	2	0.8333	0.3333	0.9333	0.6667	0.2634	1.0000	FALSE
7	3	0.8235	0.3333	0.9286	0.6905	0.3267	1.0000	FALSE
7	4	0.6667	0.6667	0.6667	0.5889	0.0634	1.0000	FALSE
7	5	0.8235	0.5000	0.8667	0.8333	0.5827	1.0000	FALSE
8	1	0.6667	0.3333	0.7333	0.6000	0.2239	0.9761	FALSE
8	2	0.7647	0.0000	0.8667	0.6667	0.3335	0.9998	FALSE
8	3	0.8235	0.3333	0.9286	0.8333	0.5943	1.0000	FALSE
8	4	0.7222	0.3333	0.8000	0.4889	0.0000	1.0000	FALSE
9	1	0.8824	0.3333	1.0000	0.4643	0.0000	0.9484	FALSE
9	2	0.7059	0.0000	0.8000	0.5333	0.0000	1.0000	FALSE
9	4	0.7222	0.0000	0.8667	0.5556	0.1417	0.9694	FALSE

9	5	0.7778	0.0000	0.9333	0.5778	0.2152	0.9403	FALSE
10	2	0.8750	0.5000	0.9286	0.8929	0.7259	1.0000	FALSE
10	3	0.6667	0.0000	0.8000	0.5444	0.1301	0.9588	FALSE
10	4	0.7778	0.6667	0.8000	0.7111	0.1418	1.0000	FALSE
10	5	0.8889	0.6667	0.9333	0.8000	0.5037	1.0000	FALSE

Table S8. Detailed training results of LR for identifying *E. coli* infection presence or absence.

Experiment Index	Fold	Accuracy	Sensitivity	Specificity	AUC	AUC_CI_low	AUC_CI_high	Is_Valid
1	2	0.9444	0.6667	1.0000	0.9333	0.7852	1.0000	TRUE
1	3	0.8824	0.6667	0.9286	0.8333	0.5943	1.0000	TRUE
2	1	1.0000	1.0000	1.0000	1.0000	1.0000	1.0000	TRUE
3	4	0.8889	0.6667	0.9333	0.9111	0.7695	1.0000	TRUE
3	5	0.8333	0.6667	0.8667	0.8889	0.7210	1.0000	TRUE
4	1	0.9412	1.0000	0.9333	1.0000	1.0000	1.0000	TRUE
5	1	0.8333	0.6667	0.8667	0.8667	0.5917	1.0000	TRUE
6	2	0.9444	0.6667	1.0000	0.9333	0.7852	1.0000	TRUE
6	3	0.8824	0.6667	0.9286	0.9048	0.7009	1.0000	TRUE
8	5	0.8333	0.6667	0.8667	0.9556	0.8501	1.0000	TRUE
9	5	0.8889	0.6667	0.9333	0.9778	0.9162	1.0000	TRUE
10	4	0.9444	0.6667	1.0000	0.8889	0.6561	1.0000	TRUE
1	1	0.8333	0.6667	0.8667	0.7333	0.2060	1.0000	FALSE
1	4	0.9412	0.5000	1.0000	0.7667	0.2910	1.0000	FALSE
1	5	0.8333	0.0000	1.0000	0.8667	0.6956	1.0000	FALSE
2	2	0.8824	0.3333	1.0000	0.7143	0.3790	1.0000	FALSE
2	3	0.7778	0.3333	0.8667	0.8222	0.6020	1.0000	FALSE
2	4	0.8333	0.3333	0.9333	0.9111	0.7695	1.0000	FALSE
2	5	0.8333	0.3333	0.9333	0.5778	0.0975	1.0000	FALSE
3	1	0.8333	0.0000	1.0000	0.5111	0.0895	0.9327	FALSE
3	2	0.9375	0.5000	1.0000	1.0000	1.0000	1.0000	FALSE
3	3	0.8333	0.3333	0.9333	0.6889	0.1953	1.0000	FALSE
4	2	0.7222	0.0000	0.8667	0.8000	0.5905	1.0000	FALSE
4	3	0.8889	0.3333	1.0000	1.0000	1.0000	1.0000	FALSE
4	4	0.8889	0.3333	1.0000	0.8444	0.6169	1.0000	FALSE
4	5	0.8235	0.3333	0.9286	0.7143	0.2139	1.0000	FALSE
5	2	0.8824	0.5000	0.9333	0.9000	0.7438	1.0000	FALSE
5	3	0.7778	0.0000	0.9333	0.9333	0.8027	1.0000	FALSE
5	4	0.8235	0.0000	1.0000	0.7381	0.4382	1.0000	FALSE
5	5	0.7778	0.3333	0.8667	0.8000	0.5482	1.0000	FALSE
6	1	0.8333	0.3333	0.9333	0.7111	0.3802	1.0000	FALSE
6	4	0.8889	0.3333	1.0000	0.9333	0.8124	1.0000	FALSE
6	5	0.8235	0.0000	0.9333	0.8000	0.5723	1.0000	FALSE

7	1	0.8889	0.3333	1.0000	0.9556	0.8582	1.0000	FALSE
7	2	0.8333	0.3333	0.9333	0.7333	0.3182	1.0000	FALSE
7	3	0.8824	0.3333	1.0000	0.8095	0.4834	1.0000	FALSE
7	4	0.8333	0.3333	0.9333	0.6333	0.0369	1.0000	FALSE
7	5	0.8824	0.5000	0.9333	0.9333	0.8027	1.0000	FALSE
8	1	0.8889	0.3333	1.0000	0.9556	0.8501	1.0000	FALSE
8	2	0.8235	0.0000	0.9333	0.8333	0.6389	1.0000	FALSE
8	3	0.8824	0.3333	1.0000	0.9762	0.9102	1.0000	FALSE
8	4	0.8333	0.3333	0.9333	0.6222	0.1085	1.0000	FALSE
9	1	0.8824	0.3333	1.0000	0.8690	0.6773	1.0000	FALSE
9	2	0.8235	0.0000	0.9333	0.9000	0.7438	1.0000	FALSE
9	3	0.8889	0.3333	1.0000	1.0000	1.0000	1.0000	FALSE
9	4	0.8333	0.0000	1.0000	0.5556	0.1417	0.9694	FALSE
10	1	0.8333	0.0000	1.0000	0.6222	0.1085	1.0000	FALSE
10	2	0.9375	0.5000	1.0000	0.8929	0.6551	1.0000	FALSE
10	3	0.8333	0.3333	0.9333	0.9222	0.7880	1.0000	FALSE
10	5	0.7778	0.0000	0.9333	0.8444	0.6560	1.0000	FALSE

Table S9. Detailed training results of RF for identifying *E. coli* infection presence or absence.

**Experiment Index**	**Fold**	**Accuracy**	**Sensitivity**	**Specificity**	**AUC**	**AUC_CI_low**	**AUC_CI_high**	**Is_Valid**
1	2	0.9444	0.6667	1.0000	0.9111	0.7205	1.0000	TRUE
2	1	1.0000	1.0000	1.0000	1.0000	1.0000	1.0000	TRUE
2	3	0.9444	0.6667	1.0000	0.9556	0.8501	1.0000	TRUE
3	2	1.0000	1.0000	1.0000	1.0000	1.0000	1.0000	TRUE
3	4	0.9444	0.6667	1.0000	0.9333	0.7852	1.0000	TRUE
3	5	0.9444	0.6667	1.0000	0.9778	0.9162	1.0000	TRUE
4	1	0.9412	1.0000	0.9333	1.0000	1.0000	1.0000	TRUE
4	3	0.9444	0.6667	1.0000	1.0000	1.0000	1.0000	TRUE
5	1	0.9444	0.6667	1.0000	0.9333	0.7852	1.0000	TRUE
5	2	1.0000	1.0000	1.0000	1.0000	1.0000	1.0000	TRUE
6	2	1.0000	1.0000	1.0000	1.0000	1.0000	1.0000	TRUE
6	3	0.9412	0.6667	1.0000	0.9048	0.7009	1.0000	TRUE
8	1	0.9444	0.6667	1.0000	0.9556	0.8501	1.0000	TRUE
8	3	0.9412	0.6667	1.0000	0.9762	0.9102	1.0000	TRUE
8	5	1.0000	1.0000	1.0000	1.0000	1.0000	1.0000	TRUE
9	3	0.9444	0.6667	1.0000	0.9778	0.9162	1.0000	TRUE
9	5	0.8889	0.6667	0.9333	0.9778	0.9162	1.0000	TRUE
10	4	0.9444	0.6667	1.0000	1.0000	1.0000	1.0000	TRUE
10	5	0.9444	0.6667	1.0000	0.8889	0.6561	1.0000	TRUE
1	1	0.8889	0.6667	0.9333	0.7556	0.2703	1.0000	FALSE
1	3	0.8824	0.3333	1.0000	0.9048	0.7473	1.0000	FALSE

1	4	0.9412	0.5000	1.0000	0.9333	0.7752	1.0000	FALSE
1	5	0.8333	0.0000	1.0000	0.9333	0.7852	1.0000	FALSE
2	2	0.8824	0.3333	1.0000	0.8571	0.5631	1.0000	FALSE
2	4	0.8333	0.0000	1.0000	0.9333	0.8124	1.0000	FALSE
2	5	0.8333	0.3333	0.9333	0.8000	0.5340	1.0000	FALSE
3	1	0.8333	0.0000	1.0000	0.7778	0.5298	1.0000	FALSE
3	3	0.8333	0.3333	0.9333	0.7778	0.5018	1.0000	FALSE
4	2	0.8333	0.0000	1.0000	0.8444	0.6560	1.0000	FALSE
4	4	0.8889	0.3333	1.0000	0.9111	0.7205	1.0000	FALSE
4	5	0.8824	0.3333	1.0000	0.8095	0.4834	1.0000	FALSE
5	3	0.8889	0.3333	1.0000	0.9556	0.8582	1.0000	FALSE
5	4	0.8235	0.0000	1.0000	0.7619	0.3492	1.0000	FALSE
5	5	0.8889	0.3333	1.0000	0.9333	0.7852	1.0000	FALSE
6	1	0.8333	0.0000	1.0000	0.6889	0.4475	0.9303	FALSE
6	4	0.8889	0.3333	1.0000	0.9333	0.8124	1.0000	FALSE
6	5	0.8824	0.0000	1.0000	0.9333	0.7752	1.0000	FALSE
7	1	0.8889	0.3333	1.0000	0.9111	0.7639	1.0000	FALSE
7	2	0.8889	0.3333	1.0000	0.6667	0.2277	1.0000	FALSE
7	3	0.8824	0.3333	1.0000	1.0000	1.0000	1.0000	FALSE
7	4	0.8333	0.0000	1.0000	0.7000	0.3969	1.0000	FALSE
7	5	0.9412	0.5000	1.0000	1.0000	1.0000	1.0000	FALSE
8	2	0.8824	0.0000	1.0000	0.8667	0.6676	1.0000	FALSE
8	4	0.8333	0.0000	1.0000	0.7111	0.4426	0.9796	FALSE
9	1	0.8824	0.3333	1.0000	0.8214	0.5868	1.0000	FALSE
9	2	0.8824	0.0000	1.0000	1.0000	1.0000	1.0000	FALSE
9	4	0.8333	0.0000	1.0000	0.8000	0.5010	1.0000	FALSE
10	1	0.8889	0.3333	1.0000	0.7333	0.2648	1.0000	FALSE
10	2	0.9375	0.5000	1.0000	0.9286	0.7593	1.0000	FALSE
10	3	0.8889	0.3333	1.0000	0.9444	0.8390	1.0000	FALSE
